# Human D-aspartate Oxidase: A Key Player in D-aspartate Metabolism

**DOI:** 10.3389/fmolb.2021.689719

**Published:** 2021-06-23

**Authors:** Loredano Pollegioni, Gianluca Molla, Silvia Sacchi, Giulia Murtas

**Affiliations:** Department of Biotechnology and Life Sciences, University of Insubria, Varese, Italy

**Keywords:** NMDA-receptor, structure-function relationships, biochemical properties, neurotranmitters, D-serine, D-aspartate

## Abstract

In recent years, the D-enantiomers of amino acids have been recognized as natural molecules present in all kingdoms, playing a variety of biological roles. In humans, d-serine and d-aspartate attracted attention for their presence in the central nervous system. Here, we focus on d-aspartate, which is involved in glutamatergic neurotransmission and the synthesis of various hormones. The biosynthesis of d-aspartate is still obscure, while its degradation is due to the peroxisomal flavin adenine dinucleotide (FAD)-containing enzyme d-aspartate oxidase. d-Aspartate emergence is strictly controlled: levels decrease in brain within the first days of life while increasing in endocrine glands postnatally and through adulthood. The human d-aspartate oxidase (hDASPO) belongs to the d-amino acid oxidase-like family: its tertiary structure closely resembles that of human d-amino acid oxidase (hDAAO), the enzyme that degrades neutral and basic d-amino acids. The structure-function relationships of the physiological isoform of hDASPO (named hDASPO_341) and the regulation of gene expression and distribution and properties of the longer isoform hDASPO_369 have all been recently elucidated. Beyond the substrate preference, hDASPO and hDAAO also differ in kinetic efficiency, FAD-binding affinity, pH profile, and oligomeric state. Such differences suggest that evolution diverged to create two different ways to modulate d-aspartate and d-serine levels in the human brain. Current knowledge about hDASPO is shedding light on the molecular mechanisms underlying the modulation of d-aspartate levels in human tissues and is pushing novel, targeted therapeutic strategies. Now, it has been proposed that dysfunction in NMDA receptor-mediated neurotransmission is caused by disrupted d-aspartate metabolism in the nervous system during the onset of various disorders (such as schizophrenia): the design of suitable hDASPO inhibitors aimed at increasing d-aspartate levels thus represents a novel and useful form of therapy.

## Introduction

**FIGURE 1:**
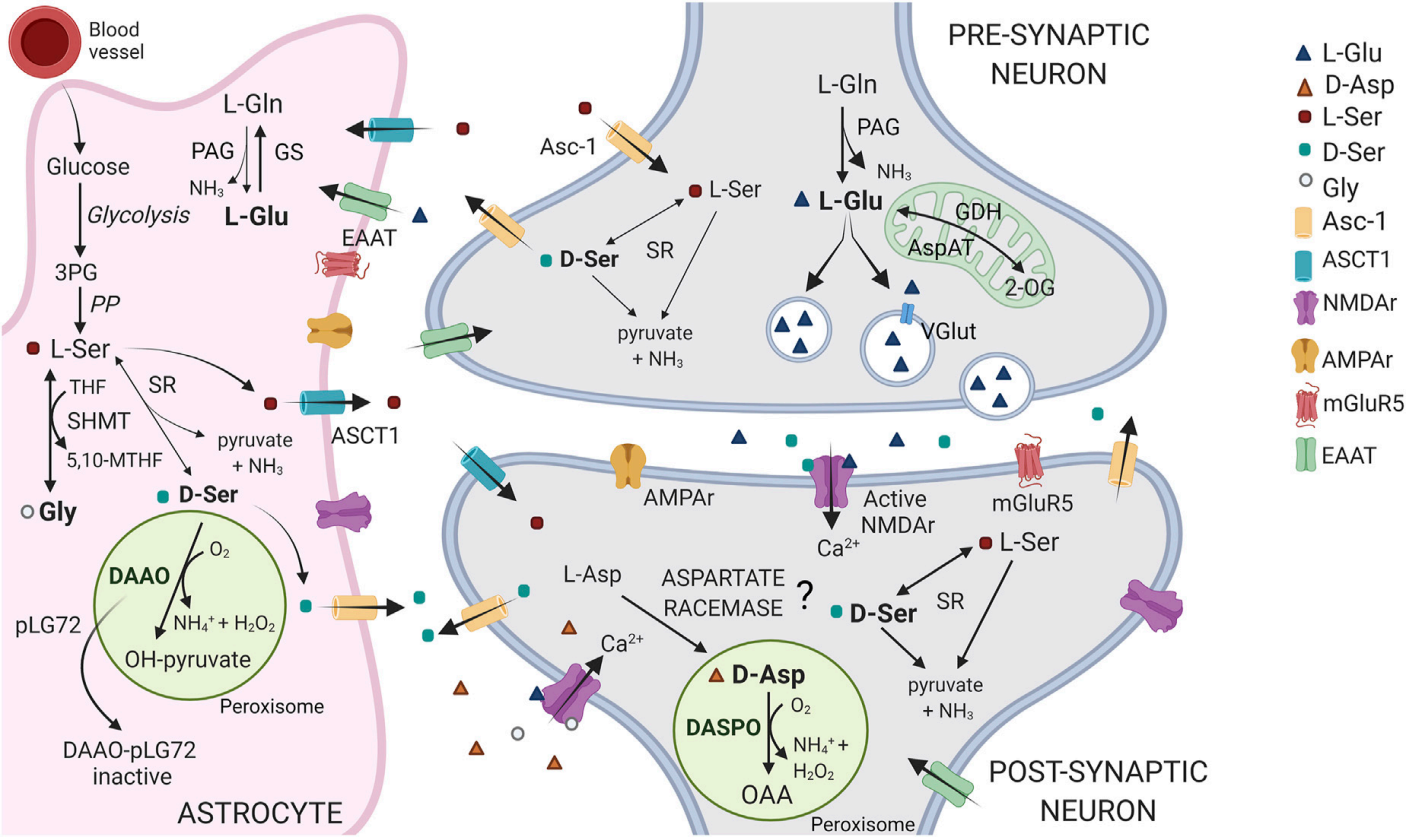
Schematic representation of the D-Asp, D-Ser and Glu pathways at tripartite synapsis. In the brain, D-Asp and DASPO are primarily present in neurons, within the peroxisomes. D-Asp biosynthetic pathway is still debated: the presence of an enzyme acting as an aspartate racemase has been proposed. L-Ser is largely synthesized in astrocytes from the glycolytic intermediate 3-phosphoglycerate (3PG), through the so called “phosphorylated pathway” (PP). L-Ser is released from astrocytes by the alanine/serine/cysteine/threonine transporter-1 (ASCT1) and is then taken up into neurons *via* transporter(s), where it is converted to D-Ser by serine racemase (SR). D-Ser is then released at the synapsis by the alanine-serine-cysteine-1 transporter (Asc-1, at least in part), where it binds to NMDA receptors at the coagonist regulatory site. Finally, it is removed from the synaptic cleft by reuptake into astrocytes where it is catabolized by DAAO, which activity is controlled by the interaction with the regulatory protein pLG72. Notably, L-Ser is also converted into glycine (the other coagonist of the NMDA receptor) by the enzyme serine hydroxymethyltransferase (SHMT). In neurons, the principal agonist of NMDA receptors, Glu is mainly produced from the reaction catalyzed by phosphate-activated glutaminase (PAG) and uploaded in vesicles through the vesicular glutamate transporter (VGlut), for the subsequent synaptic release and post-synaptic neurons stimulation. Glu signal is terminated by the uptake into astrocytes that occurs through the amino acid transporters (EEAT). In glial cells, Glu is converted to Gln *via* glutamine synthetase (GS) or 2-oxoglutarate by glutamate dehydrogenase or aspartate aminotransferase (GDH and AspAT) for subsequent oxidative metabolism in the Krebs cycle. Gln is then shuttled back to neurons. 2-OG = 2-oxoglutarate; OAA = oxaloacetate.

For a long time, the distribution and significance of d-amino acids were called into question as they were considered the “wrong” enantiomers of amino acids; we now know they are present in all organisms where they play different, specialized roles (Alieshkevich et al., 2018; Sasabe and Suzuki, 2018). In mammals, d-serine (D-Ser) and d-aspartate (D-Asp) attracted researchers’ interest most because of their involvement in physiological processes. A schematic representation of D-Ser and D-Asp metabolism and role at the tripartite synapsis is shown in Figure 1. In the past 20 years, a number of studies focused on D-Ser since, in many areas of the mature brain, it is the preferred coagonist for synaptic N-methyl-d-aspartate (NMDA) receptors, a subtype of the glutamate ionotropic receptor family. D-Ser activates the receptor by binding to the so-called “glycine-binding site” of GluN1 subunits (alternatively to glycine) and potentiates NMDA receptor-mediated responses (Panatier et al., 2006; Wolosker 2007; Henneberger et al., 2010; LeBail et al., 2015; Ferreira et al., 2017), thus playing an essential role in synaptic plasticity. Accordingly, perturbation of D-Ser levels in brain, cerebrospinal fluid (CSF), and serum has been related to the pathophysiology of various neurological and psychiatric disorders (Pollegioni and Sacchi, 2010), e.g., Alzheimer’s disease (Wu et al., 2004; Madeira et al., 2015; Piubelli et al., 2021), schizophrenia (Hashimoto et al., 2003; Bendikov et al., 2006), and amyotrophic lateral sclerosis (Sasabe et al., 2007; Sasabe et al., 2012). In most recent times, D-Asp was also widely investigated because of its role in the central nervous, neuroendocrine, and endocrine systems (Wolosker et al., 2000; Katane and Homma, 2011; Errico et al., 2012; Chieffi Baccari et al., 2020; Usiello et al., 2020).

D-Asp stimulates postsynaptic NMDA receptors by binding to the glutamate site of GluN2 subunits, Figure 1 (Errico et al., 2012; Ota et al., 2012; Errico et al., 2015). In addition, D-Asp also stimulates metabotropic glutamate receptor 5 (mGlu5) (Molinaro et al., 2010) and presynaptic *α*-amino-3-hydroxy-5-methylisoxazole-4-propionic acid hydrate (AMPA), mGlu5, and NMDA receptors (Cristino et al., 2015). Studies performed in animal models characterized by a persistent deregulation of D-Asp levels (i.e., DDO knockout or C57BL/6J mice chronically administered D-Asp) showed that it affects NMDA receptor-dependent processes such as synaptic transmission, plasticity, and cognition (Errico et al., 2008; Errico et al., 2011a; Errico et al., 2011b; Errico et al., 2014). D-Asp counteracts the decrease in NMDA receptor signaling observed during aging (Errico et al., 2011a,b), has beneficial effects during remyelination (DeRosa et al., 2019), and influences the process leading to A*β*40 and A*β*42 aggregation in Alzheimer’s disease (D’Aniello et al., 2017). Furthermore, reduced levels of D-Asp have been reported in prefrontal cortex and striatum in patients with schizophrenia (Errico et al., 2013).

In the brain, D-Asp is mainly present in neurons (Schell et al., 1997; Errico et al., 2012): its levels are highest during the embryonic phase and in the first days of life and drop gradually in adulthood (Hashimoto et al., 1993; Errico et al., 2011a; Punzo et al., 2016). In contrast, an increase in D-Asp levels is observed in endocrine glands during postnatal and adult developmental stages, which draws a parallel with the synthesis of different hormones and of melatonin (Di Fiore et al., 2014; Chieffi Baccari et al., 2020). Notably, the progressive expression of the degradative enzyme d-aspartate oxidase (DASPO or DDO, EC 1.4.3.1) during development is responsible for the observed decrease in brain D-Asp levels during the postnatal phase: in the adult brain, D-Asp is localized inversely to DASPO (Schell et al., 1997; Errico et al., 2012).

**FIGURE 2:**
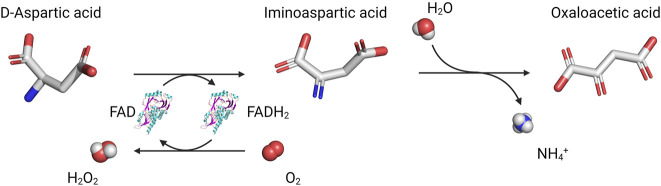
Reaction catalyzed by DASPO on acidic d-amino acids.

The source of brain D-Asp is still being debated. Acidic d-amino acids are present in different food specimens (Friedman 2010; Friedman and Levin 2012; Marcone et al., 2020): exogenous D-Asp, D-Ser and d-proline alike (Bauer et al., 2005; Langen et al., 2005) can efficiently cross the blood-brain barrier. A PLP-dependent aspartate racemase that reversibly converts L- into D-Asp was identified in the rat pituitary gland (Wolosker et al., 2000; Topo et al., 2009), thyroid, and testis (Topo et al., 2009; Topo et al., 2010); however, its relevance in mammals is still unclear (Kim et al., 2010; Matsuda et al., 2015; Tanaka-Hayashi et al., 2015). As little is known about the biosynthetic pathway, the only way to control D-Asp levels in the brain is to modulate its degradation. In mammalian tissues, three enzymes able to stereoselectively degrade d-amino acids have been identified, namely, d-amino acid oxidase (DAAO or DAO, EC 1.4.3.3), DASPO, and d-glutamate cyclase (EC 4.2.1.48) (Pollegioni et al., 2007; Katane and Homma, 2010; Takahashi 2020), the latter metabolizing d-glutamate (D-Glu), but not D-Asp in mouse heart (Ariyoshi et al., 2017; Tateishi et al., 2017). DAAO and DASPO, discovered by Krebs in 1935 (Krebs 1935), are peroxisomal flavoproteins (Usuda et al., 1986; van Veldhoven et al., 1991; Zaar 1996; Zaar et al., 2002) that catalyze the oxidative deamination of d-amino acids into the corresponding *α*-ketoacids and ammonia; the reduced FADH_2_ cofactor is regenerated by dioxygen, yielding hydrogen peroxide (Figure 2). DAAO oxidizes several neutral and basic d-amino acids (including D-Ser): the human enzyme (hDAAO) shows peculiar properties such as low kinetic efficiency and weak flavin binding (Molla et al., 2006; Caldinelli et al., 2009; Sacchi et al., 2012). In contrast, DASPO is highly specific for acidic d-amino acids only (Katane et al., 2015a; Molla et al., 2020; Takahashi 2020), which are not oxidized by DAAO; the human DASPO (hDASPO) shows a high turnover and tight cofactor binding. DAAO and DASPO share a high sequence identity; thus, it has been proposed that they derive from a common ancestor (Negri et al., 1992; Takahashi et al., 2004), but seemingly evolved to fulfil different and specific physiological roles.

Here, we review the main properties of hDASPO and current knowledge regarding the regulation of enzyme activity and expression, with the aim of providing a useful tool to comprehend the molecular mechanisms underlying the modulation of D-Asp levels in human tissues and to push novel therapeutic strategies targeting such a valuable target.

## Human D-aspartate Oxidase Isoforms

The UniProtKB/Swiss-Prot database (www.uniprot.org/help/uniprotkb) predicts four different splice isoforms for the human *DDO* gene transcript, referred to as DDO-1–4; in contrast, no splice variants for the homologous *DAO* gene are reported.

DDO-1 represents the canonical isoform and encodes for a 341 amino acid protein (Q99489-1, 37.5 kDa; hDASPO_341 referred to as the wild-type sequence), the one that has been investigated most; see below (Setoyama and Miura, 1997; Molla et al., 2020; Rabattoni et al., 2021).

**FIGURE 3:**
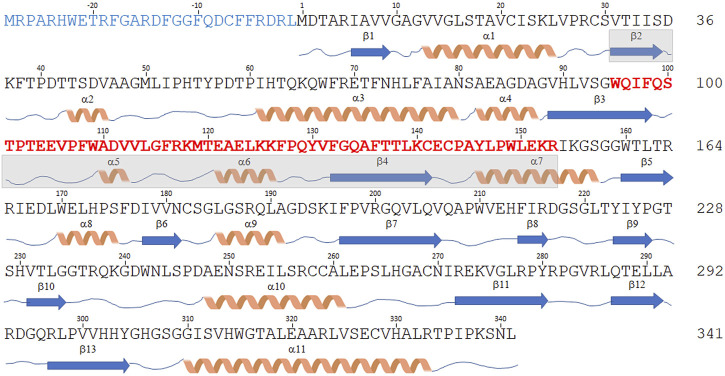
Sequence comparison of hDASPO isoforms. Schematic representation of the amino acid sequence of the hDASPO isoforms reported in the UniProtKB/Swiss-Prot database. The secondary structure elements (as determined for the canonical hDASPO_341) are reported below. The N-terminal 28 additional residues of the hDASPO_369 isoform are depicted in blue. The 59 amino acids long portion of the protein lacking in the hDASPO_282 isoform are depicted in bold red (and the corresponding secondary structure elements are shaded in cyan).

The DDO-2 isoform is identical to the canonical one but lacks the central region of the transcript (encoding for residues 95–153), due to the fact that exon 4 is skipped in the original transcript sequence (Figure 3). The expression of this variant, which is 59 residues shorter (282 amino acids, 30.5 kDa), in *E. coli* yielded an accumulation of the recombinant protein as inclusion bodies (Setoyama and Miura, 1997); therefore, it was not characterized.

The DDO-3 isoform differs from the canonical one since it encodes a protein with 28 additional N-terminal residues (369 amino acids, 41 kDa, hDASPO_369, Figure 3), an upstream alternative start codon being recognized in the primary transcript. Notably, a BLAST search reveals that this long form is highly conserved in primates: homologous forms of mouse and beef DASPOs (albeit as partial sequences) are reported in the NCBI protein database (www.ncbi.nlm.nih.gov/protein). Worthy of note, we found very recently that the DDO-3 long isoform was differentially expressed in a cohort of individuals with Alzheimer’s disease. Mass spectrometry analysis identified its N-terminal peptide in the hippocampus of female patients only, while peptides corresponding to regions common to hDASPO_341 and _369 protein isoforms were detected in samples from male and female patients and healthy subjects (Rabattoni et al., 2021). Compared to the canonical shorter isoform, hDASPO_369 is characterized by a very low solubility when overexpressed in *E. coli*: this hampered the in-depth investigation of its biochemical properties. Nevertheless, by using a transfected U87 (human glioblastoma) cell line ectopically expressing this protein variant we observed that the additional N-terminal sequence does not affect enzyme function and cellular localization. Similar to hDAAO, both the long and the canonical hDASPO isoforms were very stable (estimated half-life ∼ 100 h). However, unlike hDAAO which is mainly degraded through the lysosome/endosome pathway (Cappelletti et al., 2014), both hDASPO variants were degraded by the ubiquitin-proteasome system upon ubiquitination (Rabattoni et al., 2021).

Finally, the DDO-4 transcript isoform combines features of the DDO-2 and DDO-3 isoforms: it encodes a 310 amino acid protein (34 kDa, Figure 3) containing the additional N-terminal residues of hDASPO_369 and lacking the 59-residue-long central portion. No attempts to heterologously express this protein have been undertaken so far.

## Biochemical Properties

hDASPO_341 (the canonical isoform) was first produced in recombinant form in *E. coli* in 1997 with a yield of 2 mg/L and partially characterized in 2015 (Setoyama and Miura, 1997; Katane et al., 2015a). More recently, the production procedure was optimized (up to 30 mg/L) so that the biochemical properties of the enzyme could be comprehensively characterized and its structure-function relationships elucidated (Molla et al., 2020).

hDASPO belongs to the family of FAD-containing oxidoreductases and shows the typical properties of this group of proteins (Molla et al., 2020): the canonical absorbance peaks at 280, 370 and 455 nm in the oxidized form; the ability to stabilize the anionic semiquinone form of the cofactor and to covalently bind sulfite; the rapid conversion of the oxidized into the reduced form by adding the substrate under anaerobic conditions; and the high reactivity of the reduced enzyme form with molecular oxygen.

hDASPO shows good activity and stability in the 8–12 pH range (Katane et al., 2015a), differently from porcine kidney DASPO (pkDASPO, where maximal activity is constant between pH 7.5 and 9) (Yamamoto et al., 2007) and mouse DASPO (mDASPO, in which activity is constant between pH 4 and 10) (Puggioni et al., 2020). The human flavoenzyme is fully stable up to 45°C, a temperature corresponding to the optimum for its enzymatic activity. The melting temperature determined following the loss of activity is 55°C (Katane et al., 2015a), higher than the value obtained following the CD signal at 222 nm (48.8°C) (Molla et al., 2020), this suggesting that the alteration in secondary structure leads to the loss of enzymatic activity. Both human and mouse DASPOs are stabilized by the presence of ligands in the active site (Molla et al., 2020; Puggioni et al., 2020).

As the bovine counterpart, the holoenzyme form of recombinant hDASPO is a monomer with a molecular mass of ∼40 kDa. Differently, the apoprotein form is present in solution as an equilibrium between monomeric and trimeric species (Molla et al., 2020). Other mammalian DASPOs have been reported to be dimeric or tetrameric (Katane et al., 2018; Puggioni et al., 2020).

The binding of the FAD cofactor to hDASPO is characterized by a tight interaction (K_d_ ∼ 33 nM) (Molla et al., 2020), similar to that observed for bovine kidney (bkDASPO) (Negri et al., 1987) and rat (rDASPO) DASPOs (Katane et al., 2018). Due to the high affinity for the flavin cofactor, hDASPO is fully present in solution as an active holoenzyme (Molla et al., 2020), whereas a different situation is apparent for mDASPO, which shows a 100-fold higher K_d_ value for the cofactor, suggesting that at physiological FAD concentration (approximatively 2.5 *μ*M in murine brain) (Decker and Byerrum, 1954) this enzyme should be present in solution in equilibrium between the active and the inactive forms (Katane et al., 2018). The interaction with the cofactor modulates hDASPO conformation (altering both the secondary and the tertiary structure) and stabilizes the protein (increasing the melting temperature of ∼7°C) (Molla et al., 2020).

Concerning the substrate specificity, different results are reported in the literature, probably due to the different experimental conditions and assays used. All mammalian DASPOs are highly specific for acidic d-amino acids. hDASPO shows the highest activity on D-Asp and NMDA, followed by D-Glu and D-Asn (13 and 10% of the value assayed on D-Asp) and shows negligible activity on D-His and D-Pro (1% vs. D-Asp) (Molla et al., 2020). pkDASPO and bkDASPO prefer NMDA to other acidic D-AAs (Table 1). All mammalian DASPOs show similar apparent K_m_ values for D-Asp, the only exception being mDASPO, which shows a higher figure. The apparent k_cat_ and catalytic efficiency values for hDASPO are significantly higher than those of all mammalian DASPOs (Table 1), i.e., hDASPO shows a 5-fold higher specific activity on D-Asp than the bovine enzyme.

**TABLE 1:** Comparison of apparent kinetic parameters of mammalian DASPOs on selected substrates (determined at 21% oxygen saturation). In parenthesis is reported the specific activity value (as U/mg protein).

	D-Asp			NMDA			D-Glu			D-Asn		
	K_m_ (mM)	k_cat_ (s^−1^)	k_cat_/K_m_ (s^−1^ mM^−1^)	K_m_ (mM)	k_cat_ (s^−1^)	k_cat_/K_m_ (s^−1^ mM^−1^)	K_m_ (mM)	k_cat_ (s^−1^)	k_cat_/K_m_ (s^−1^ mM^−1^)	K_m_ (mM)	k_cat_ (s^−1^)	k_cat_/K_m_ (s^−1^ mM^−1^)
hDASPO	1.05^a^	81.3^a^	77.4^a^ (124)^a^	2.8^a^	73.6^a^	26.7^a^ (112)^a^	31.5^a^	11.3^a^	0.36^a^ (17.3)^a^	6.7^a^	8.3^a^	0.12^a^ (12.7)^a^
	2.1^b^	68.4^b^	32.7^b^ (87.5)^b^	6.8^c^	37.7^c^	5.5^c^ (91.2)^c^	—	—	—	—	—	—
	2.7^c^	52.5^c^	19.0^c^ (77.4)^d^	—	—	—	—	—	—	—	—	—
rDASPO	2.3^b^	31.1^b^	14.1^b^ (71.7)^d^	—	—	—	—	—	(1.8)^d^	—	—	—
mDASPO	7.4^b^	9.8^b^	1.35^b^ (12.2)^b^	4.2^e^	112.1^e^	26.7^e^ (172)^e^	68.4^e^	16.8^e^	0.25^e^ (25.7)^b^	101.4^e^	11.4^e^	0.4^e^ (17.4)^e^
	2.1^e^	58.4^e^	27.8^e^ (89.4)^e^	9.7^f^	13.0^f^	1.3^f^ (13.1)^b^	79.4^f^	1.6^f^	0.02^f^ (0.5)^b^ ^,^ ^d^	—	—	(0.2)^b^
	8.8^f^	12.2^f^	1.4^f^	—	—	—	—	—	—	—	—	—
pkDASPO^g^	2.5	37.5	14.8 (62.0)	0.9	45.1	52.9	66.9	9.4	0.1	—	—	—
bkDASPO^h^	3.7	22.5	6.1 (25.3)^i^	1.5	30.9	20.6	5.6	1.2	0.2	—	—	—

a Molla et al., 2020.

b Katane et al., 2015a.

c Setoyama et al., 1997.

d Katane et al., 2018.

e Puggioni et al., 2020.

f Katane et al., 2011.

g Yamamoto et al., 2007.

h Negri et al., 1988.

i Negri et al., 1987.

The oxidative deamination of acidic d-amino acids catalyzed by hDASPO follows a ternary-complex mechanism in which the complex between the reduced flavin and the imino acid reacts with oxygen before the imino acid is released (Molla et al., 2020). During the reductive half-reaction, substrate dehydrogenation proceeds by direct transfer of a hydride from the *α*-carbon of the substrate to the flavin N5, as elegantly demonstrated for DAAO by Sandro Ghisla’s lab (Pollegioni et al., 1997; Umahu et al., 2000; Harris et al., 2001; Saam et al., 2010). Conversion of the d-amino acid into the planar imino acid together with the flavin reduction is very fast and seems to be reversible, with an equilibrium constant of ∼5 for the overall process (Molla et al., 2020). The rate constant for flavin reduction by D-Asp (k_red_ value estimated ∼1,550 s^−1^) is higher than the k_cat_ value (230 s^−1^), suggesting that substrate oxidation is not the rate-limiting step during catalysis. However, the high apparent K_d_ for D-Asp (23 mM) indicates that the substrate binding largely controls the reaction rate. Differently from bkDASPO (in which the rate-limiting step is represented by a conformational change related to the binding of a second molecule of D-Asp to the reduced enzyme) (Negri et al., 1988), the rate-determining step in hDASPO is represented by the reoxidation of the reduced flavin. This step corresponds to a single exponential process with a rate constant of ∼1 × 10^5^ M^−1^ s^−1^ (Molla et al., 2020).

## Structural Properties

The 3D structure of hDASPO was solved at 3.2 Å resolution from the diffraction data collected for the C141Y/C143G variant; these substitutions are present in the pkDASPO sequence and do not alter the functional and structural properties of the enzyme. The structure was solved by molecular replacement using the coordinates of hDAAO as starting model (pdb 3g3e); electron density at the active site was modeled as a glycerol molecule, a component of the protein buffer (Molla et al., 2020).

**FIGURE 4:**
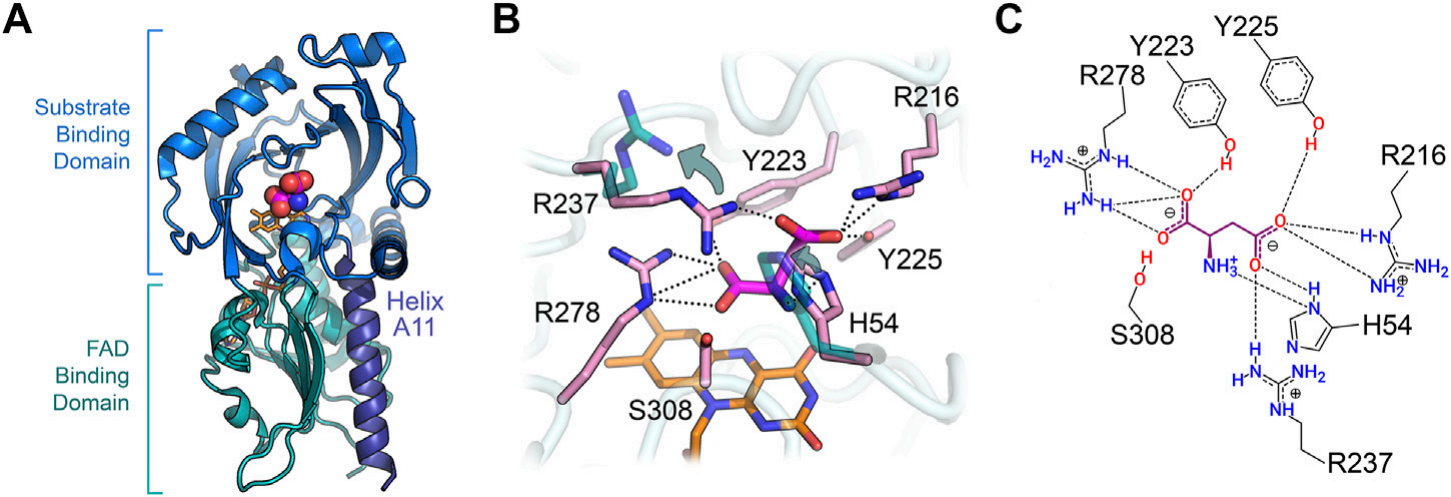
Three-dimensional structure of human DASPO (PDB entry 6rkf). **(A)** Overall fold of the hDASPO monomer. The FAD binding domain is shown in teal and the substrate binding domain is shown in blue. The substrate D-Asp, docked at the active site (Molla et al., 2020), is shown with a sphere representation. The FAD cofactor is in orange and *α*-helix A11 is depicted in violet. **(B)** Close up of the active site. The side chains of residues Arg237 and His54 in the alternative open conformation are shown in teal (see arrows); non-covalent interactions between D-Asp and the active site residues are shown as dotted lines. **(C)** Scheme of the interaction of D-Asp at the active site: H-bonds are shown as dotted lines.

hDASPO belongs to the DAAO-like family of the *α*,*β*-protein class according to the SCOP classification system (SCOP family 4,000,124, class 1,000,002) and to the Pfam family of the FAD-dependent oxidoreductases (Pfam: PF01266) (Andreeva et al., 2014; Mistry et al., 2021). According to DALI analysis, the overall tertiary structure of hDASPO is similar to that of FAD-dependent oxidases active on amino acids or amines: the closest proteins are hDAAO (RMSD = 1.4 Å), glycine oxidase (RMSD = 2.8 Å), and sarcosine oxidase (chain B, RMSD = 2.5 Å) (Holm, 2020), Table 2. The tertiary structure of hDASPO can be divided into two large domains, each formed by noncontiguous sequence regions: the FAD-binding domain (FBD) and the substrate-binding domain (SBD), Figure 4A. The FBD possesses the canonical dinucleotide binding fold (i.e., the Rossman fold containing the corresponding Wierenga consensus sequence at the N-terminus) and an alternative of the Peroxisomal Targeting Signal one sequence (Ser-Asn-Leu, PROSITE, PS00342) at the C-terminus (Wierenga et al., 1986; Setoyama and Miura, 1997). The SBD is characterized by a large mixed *β*-sheet formed by eight *β*-strands, a fold similar to that observed in other amino acid oxidases (Fraaije and Mattevi, 2000; Umhau et al., 2000; Mortl et al., 2004).

**TABLE 2:** Structural comparison of protein homologous to hDASPO identified by DALI server.

PDB code	Protein	Source	DALI rank	RMSD (Å)	Aligned residues	Identity (%)
5zja	d-Amino acid oxidase	*Homo sapiens*	2	1.4	327	41
7ct4C^a^		*Rasamsonia emersonii*	3	1.8	317	33
1c0l		*Rhodotorula gracilis*	4	2.1	315	29
4ysh	Glycine oxidase	*Geobacillus kaustophilus*	5	2.8	310	21
1y56B^a^	Sarcosine oxidase	*Pyrococcus horikoshii OT3*	7	2.5	305	14
3ad7B^a^	(α subunit)	*Corynebacterium* sp. U-96	10	2.6	307	12
5fjm	L-Amino acid deaminase	*Proteus myxofaciens*	13	2.7	303	12
1pj6	N,N-Dimethylglycine oxidase	*Arthrobacter globiformis*	17	2.9	306	16
2gb0	Monomeric sarcosine oxidase	*Bacillus* sp. B-0618	21	3.0	305	13
5tti	Hydroxybenzoate hydroxylase	*Pseudomonas putida* KT2440	48	3.5	244	12
2z5x	Monoamine oxidase A	*Homo sapiens*	73	3.7	248	11
1gpe	Glucose oxidase	*Penicillium amagasakiens*	106	4.5	262	10
2e5v	L-Aspartate oxidase	*Sulfolobus tokodaii*	108	3.5	211	14
3b3r	Cholesterol oxidase	*Streptomyces* sp. SA-COO	116	3.9	223	13

a The last letter identifies the chain of the corresponding 3D structure.

The FAD cofactor binds to hDASPO in an extended conformation, with the isoalloxazine ring located at the interface between the FBD and the SBD (Molla et al., 2020); this conformation is typical of flavoproteins belonging to the glutathione reductase two family (Dym and Eisenberg, 2001). The cofactor is kept in place by several electrostatic or polar interactions (mainly H-bonds) formed with atoms of 35 residues of the protein (located within 4.5 Å from the FAD molecule). In particular, the isoalloxazine ring interacts with the protein by five electrostatic interactions (with the side chain of Ser312 and the backbone of Ala48, Met50, and Ser308) and two van der Waals contacts (with Val203 and Gly276) (Molla et al., 2020). The positive N-terminal dipole of the *α*-helix A11, belonging to the FBD, points toward the O2 position of the pyrimidine ring of the isoalloxazine and stabilizes the negative charge of the reduced cofactor delocalized in this region during catalysis.

**FIGURE 5:**
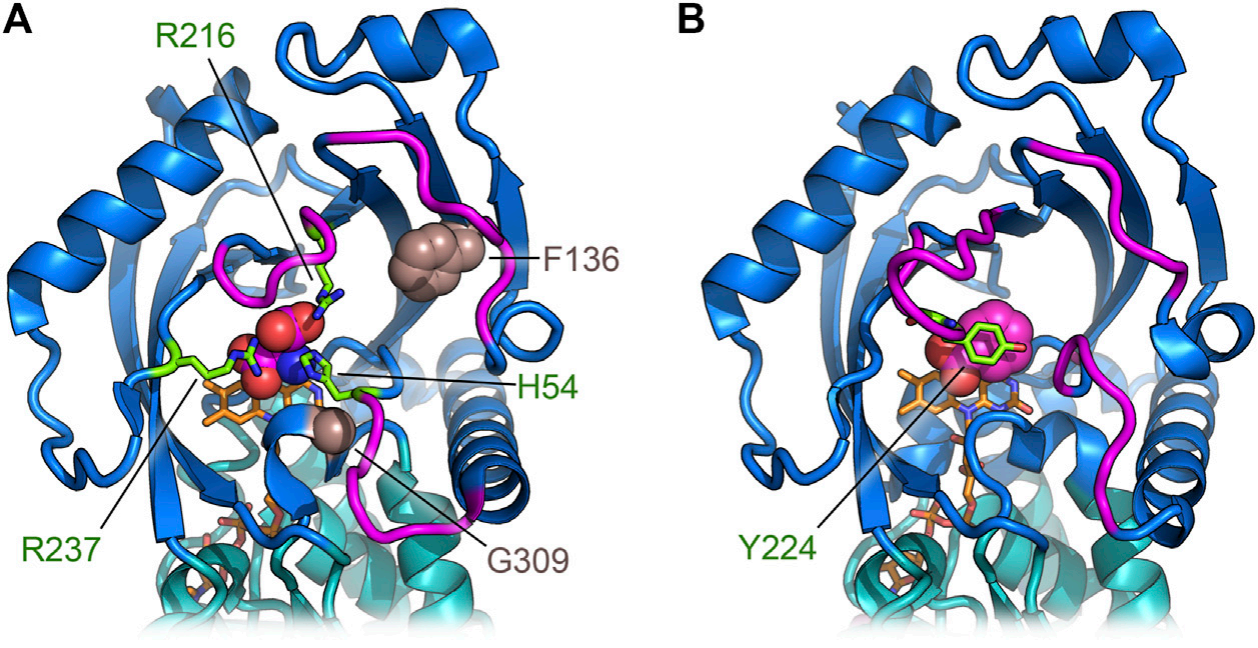
Comparison of the regions deputed to control the active site entrance in hDASPO **(A)** and hDAAO **(B)**. The loops close to the active site entrance are shown in magenta; in hDASPO the residues Phe136 and Gly309, corresponding to positions related to somatic mutation in cancer, are shown as sphere; FAD cofactor is shown in orange.

hDASPO shares the overall mode of substrate binding with the other flavin oxidases active on amino acids (e.g., DAAO, LAAO and L-amino acid deaminase) (Pollegioni et al., 2013; Molla et al., 2017; Ball et al., 2018). The active site is located in a deep cavity and is characterized by three positively charged arginine side chains (Arg216, Arg237, and Arg278) surrounding the substrate D-Asp, thus forming a tight network of electrostatic and H-bond interactions (Figures 4B,C). Arg278 forms a two-point interaction with the *α*-COOH group of the substrate: this essential residue is conserved in all known oxidases active on amino or hydroxy acids (Molla et al., 2000; Umena et al., 2006; Sacchi et al., 2012). Arg216 forms a salt bridge with the *γ*-COOH group of the side chain of D-Asp: it is fundamental in shaping the substrate scope of hDASPO. As a consequence, hDASPO shows a very narrow substrate specificity in comparison with other amino acid oxidases (Job et al., 2002; Rosini et al., 2017; Molla et al., 2020). The role of Arg216 in substrate selectivity is also supported by the biochemical properties of the R216Q hDASPO variant, which shows a lower kinetic efficiency on D-Asp and gains the ability to oxidize D-Ala (Katane et al., 2017). The third arginine (Arg237) is located at the entrance of the active site, close to His54. These two residues, whose side chains show multiple conformations in the hDASPO molecules of the crystal asymmetric unit, play three fundamental roles: i) they act as an active site gate switching between an open and a close conformation, thus affecting the kinetics of substrate binding and product release during turnover (Figure 5); ii) in the open conformation, they form a surface cluster of positive charges (together with Arg216) that attracts the negatively charged D-Asp substrate (Katane et al., 2011; Molla et al., 2020); and iii) in the close conformation, they contribute to bind the substrate D-Asp: their replacement with alanine residues resulting in a decrease in kinetic efficiency of the enzyme (in particular, a ∼20-fold increase in the apparent K_m_ for D-Asp was observed for the R237A variant) (Molla et al., 2020).

The residues Tyr223 and Tyr225 complete the tight network of interactions between the substrate D-Asp and the residues of the active site (Figures 4B,C).

## Inhibitors

In principle, it should be possible to modulate levels of D-Asp in the central nervous system by blocking hDASPO using selective inhibitors. Here, activating NMDA receptor function by increasing D-Asp levels may provide a novel therapeutic approach for psychiatric diseases such as schizophrenia.

hDASPO binds several carboxylic acids. The lowest inhibition constant (K_i_) value was determined for malonate, followed by meso-tartrate (both compounds act as substrate-competitive inhibitors) and aminooxyacetic acid, Table 3 (Katane et al., 2013; Katane et al., 2015a). Notably, the K_i_ values for rodents DASPO are significantly different from those for the human counterpart. 5-Aminonicotinic acid (5-An) efficiently inhibits hDASPO, with a K_i_ of 3.8 *μ*M (Katane et al., 2010) and a K_d_ of 10.1 *μ*M (Molla et al., 2020). hDASPO also binds known hDAAO inhibitors, such as benzoate, anthranilate, 6-chloro-1,2-benzoxazol-3-one (CBIO), and pyrido (2,3-b) pyrazine-2,3 (1H,4H) -dione (DPPD), Table 3 (Molla et al., 2020), but with a lower affinity (K_d_ values are 30-1000-fold lower for hDAAO) (Ferraris et al., 2008; Caldinelli et al., 2010; Sacchi et al., 2013). The binding mode of 5-An, CBIO, and DPPD reported in (Molla et al., 2020) highlighted how the well-known conformational change of the flexible lid at the entrance of the hDAAO active site is not observed in hDASPO because such a structural element is absent (see section “DASPO vs. DAAO”). The hDAAO inhibitor 3-hydroxyquinolin-2(1H)-one (Duplantier et al., 2009) also inhibits hDASPO activity with an IC_50_ value of 0.86 *μ*M (at 0.25 mM D-Asp concentration), corresponding to a theoretical K_i_ value of 0.76 *μ*M, Table 3 (Katane et al., 2015b).

**TABLE 3:** Inhibitors of hDASPO: inhibition and binding constants.

Compound	K_i_	IC_50_	K_d_	Reference
		(*µ*M)		
Malonate	153 ± 26	—	—	Katane et al. (2013)
*meso*-Tartrate	661 ± 48	—	157 ± 3	Katane et al. (2013); Molla et al. (2020)
L-(+)-Tartrate	—	—	870 ± 200	Molla et al. (2020)
Aminooxyacetate	1,492 ± 693	—	—	Katane et al. (2013)
5-An	3.80 ± 0.96	21.9 ± 5.6	10.1 ± 2.4	Katane et al. (2015a); Molla et al. (2020)
DPPD	—	—	31.5 ± 7.5	Katane et al. (2013)
Benzoate	—	—	7,800 ± 600	Molla et al. (2020)
Anthranilate	—	—	8,800 ± 1,500	Molla et al. (2020)
CBIO	—	—	51 ± 5	Molla et al. (2020)
TLM		∼5,000	—	Katane et al. (2010)
HHTPC	15.1 ± 2.8	87.1 ± 16.0	—	Katane et al. (2015b)
3-Hydroxyquinolin-2(1H)-one	0.76^a^	0.855	—	Duplantier et al. (2009); Katane et al. (2015b)
Olanzapine	—	23.4 ± 1.6	—	Sacchi et al. (2017)

a Calculated.

In the search for potent hDASPO inhibitor(s), biologically active compounds of microbial origin were screened by (Katane et al., 2010): this analysis identified thiolactomycin (TLM) as a mixed type inhibitor, showing an IC_50_ value of ∼5 mM for hDASPO. Furthermore, a huge number of compounds were screened in silico by a computer ligand-docking method and experimentally evaluated *in vitro* by (Katane et al., 2013; Katane et al., 2015b). The best inhibitors, namely, 5-An and 7-hydroxy-4-hydro-1,2,4-triazolo (4,3-a) pyrimidine-6-carboxylic acid (HHTPC), acting as competitive and selective inhibitors for hDASPO (since they do not affect hDAAO activity), may serve as lead compounds for the development of clinically useful hDASPO inhibitors.

We recently demonstrated that olanzapine, a commonly used second-generation antipsychotic drug, inhibits hDASPO activity: an IC_50_ value of 23.4 *μ*M was determined, a figure not affected by FAD concentration (at 4 vs. 20 *μ*M) (Sacchi et al., 2017). Additional first-generation (chlorpromazine and haloperidol) and second-generation (clozapine) antipsychotics and antidepressants (amitriptyline, bupropion and fluoxetine) did not affect hDASPO activity. The chronic administration of olanzapine (5 mg/kg) increased extracellular D-Asp levels in the prefrontal cortex of freely moving mice while the chronic administration of 5 mg/kg clozapine did not. A significant increase in extracellular D-Asp levels was apparent in control mice chronically treated with 5 mg/kg olanzapine while no change was apparent in *DDO* ^*−/−*^ mice (Sacchi et al., 2017).

## Tissue and Cellular Localization

DASPO is ubiquitously expressed in mammals: the highest amounts of the enzyme have been detected in kidney, liver, and CNS (Hamilton 1985; van Veldhoven et al., 1991); interestingly, the same distribution was reported for the orthologous DAAO (Pollegioni et al., 2007 and references therein). The presence and the physiological role of these two flavoenzymes in the brain prompted the investigation of their localization in different brain areas, tissues and cell populations.

DASPO distribution in the human CNS was first investigated by the group of Dariush Fahimi (Zaar et al., 2002). hDASPO was shown to occur in several brain regions and, differently from hDAAO which was mainly reported in glial cells (Verrall et al., 2007; Sasabe et al., 2014), to be dominantly expressed in neurons. hDASPO was widely distributed in cerebral cortex, hippocampus, diencephalon, brainstem, cerebellum, spinal cord, choroid plexus, and striatum (although here only in a small population of magnocellular neurons). Conversely, based on studies in rodents, the mammalian DAAO has been traditionally considered as a hindbrain enzyme, highly expressed in the cerebellum, spinal cord, and brainstem (Horiike et al., 1994; Kapoor and Kapoor, 1997). Only recently was hDAAO discovered to be more widespread than expected when its presence and activity (albeit at quite low levels) were detected in forebrain regions (Verrall et al., 2007 and, 2010; Madeira et al., 2008; Sasabe et al., 2014). Benzoate treatment of mild cognitive impairment patients was recently studied by resting-state functional magnetic resonance imaging scans and regional homogeneity (ReHo) analysis: the change in working memory positively correlated with decreased ReHo in right precentral gyrus and right middle occipital gyrus (Lane et al., 2021). This paper explored regional relationships between hDAAO inhibition and NMDA receptor activity enhancement in brain.

**FIGURE 6:**
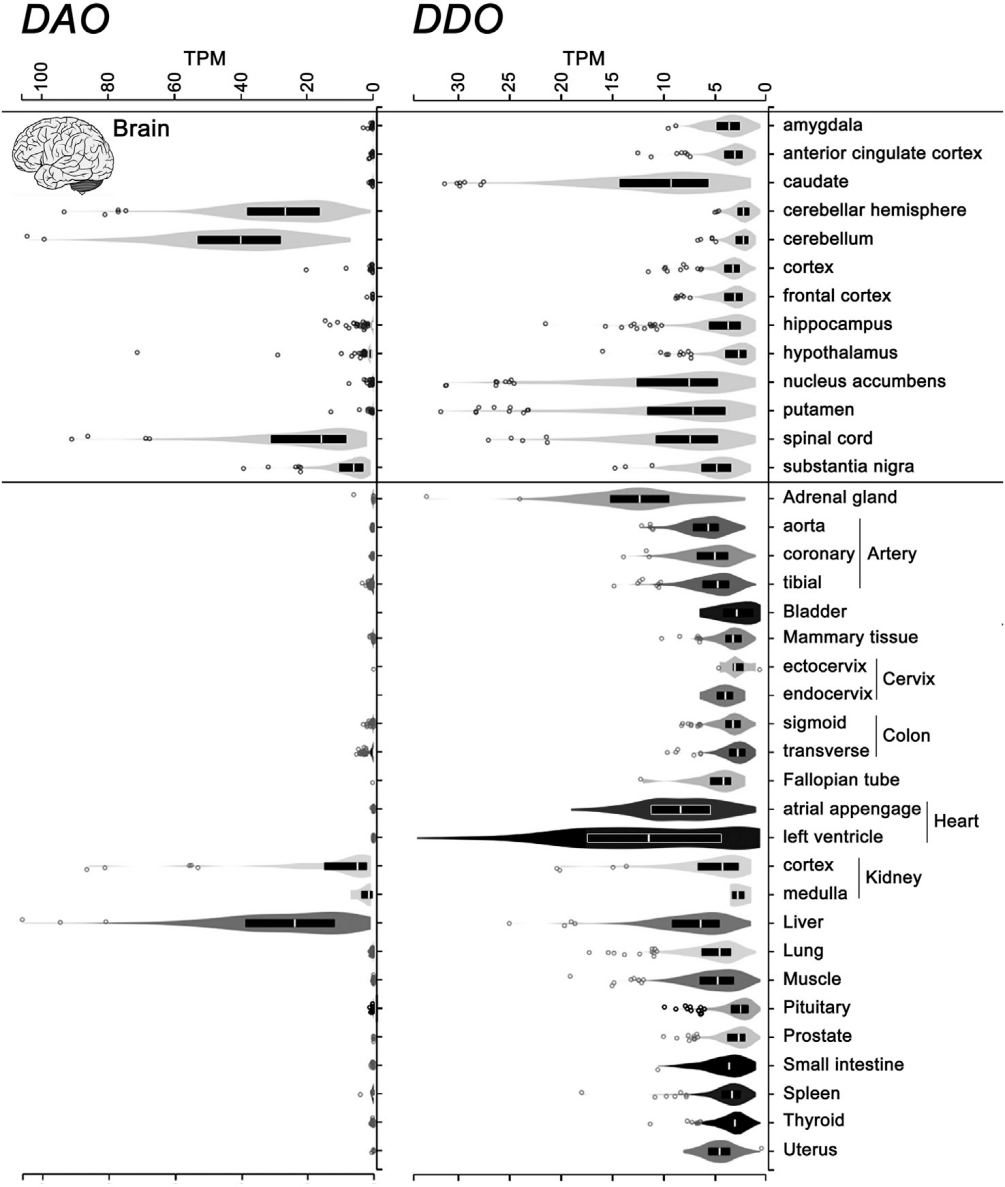
Levels of the transcripts encoding hDASPO and hDAAO in human tissues. Violin plot representing *DDO* (ENSG00000203797.9) and *DAO* (ENSG00000110887.7) gene transcript levels in different human tissues. Data were obtained from the Genotype-Tissue Expression (GTex) Project Portal, GTEx Analysis Release V8 (dbGaP Accession phs000424.v8.p2, www.gtexportal.org).Values are shown in TPM (transcripts per kilobase million); tissues in which *DDO* transcript values were lower than 2 TPM were omitted. Box plots represent the median and 25th and 75th percentiles. Dots are data points above or below 1.5-fold the interquartile range, displayed as outliers.

Differently from DAAO, the mammalian DASPO was detected in the endocrine system as well (pineal and pituitary glands, adrenal gland, thyroid gland, and testis), where it has been suggested to be involved in D-Asp homeostasis during adulthood and in hormone maturation (Usiello et al., 2020). The widespread expression of the *DDO* gene encoding hDASPO was confirmed by consulting the GTEx Portal (www.gtexportal.org): the highest transcript levels are reported in adrenal gland, heart, brain (in particular, basal ganglia and spinal cord, while very low levels are indicated in cerebellum, hypothalamus, and brain cortex), and liver, while low levels are apparent in kidney. Low but significant levels of hDASPO transcript are also reported in female reproductive organs (but not in male ones), lung, small intestine, and spleen (Figure 6). At the transcript level, in contrast, hDAAO is essentially expressed in the cerebellum, spinal cord, liver, and kidney and is generally more abundant than hDASPO.

At the protein level, the Human Proteins Atlas (www.proteinatlas.org) further highlights the extremely different pattern of expression and tissue distribution between the two amino acid oxidases.

## Regulation of Expression

The mammalian DASPO displays a peculiar temporal distribution pattern in the brain: studies in rat showed that protein expression and enzymatic activity are nearly absent during embryonic development, but sharply increase after birth, reaching significant expression levels during adulthood (van Veldhoven et al., 1991). In particular, postnatal expression of the enzyme is regulated at the transcriptional level by epigenetic events: the dynamic changes in *DDO* gene transcription are closely associated with a progressive demethylation of its putative promoter region, at eight CpG residues/islands surrounding the transcription start site (Punzo et al., 2016; Errico et al., 2020). Other work conducted at Usiello’s laboratory further explored the methylation state of the *DDO* gene promoter among different cell types and brain areas, at various developmental stage in mouse and, by analyzing specific combinations of methylated CpG sites (defined as epialleles), highlighted that neurons, oligodendrocytes, astrocytes, and microglial cells display a cell type-specific methylation pattern (Florio et al., 2017). Ultradeep methylation analysis of the mouse *DAO* gene promoter region identified dynamic demethylation at two CpG sites only in the cerebellum and in a specific time window (P1-P15) (Cuomo et al., 2019), and a specific and reproducible rearrangement of the epiallele frequency distribution in undifferentiated embryonic mouse stem cells upon neural differentiation (Florio et al., 2017).

The status of *DDO* promoter methylation and corresponding transcription levels were also investigated in the human brain (dorsolateral prefrontal cortex, hippocampus, and cerebellum of healthy subjects and schizophrenia-affected individuals) (Keller et al., 2018), showing a tissue-specific distribution of methylated CpGs with no alterations in the detected profile between diagnosis groups. The same study reported a high methylation of the *DAO* gene promoter region in all the investigated brain areas, consistent with the very low expression of hDAAO in the cortex and hippocampus, but clashing with the high expression levels detected in the cerebellum. Furthermore, the different brain areas are characterized by distinctive methylation signatures in both healthy and schizophrenic subjects, possibly reflecting differences in epiallele distribution among cells and a specific CpG combinatorial code (Keller et al., 2018).

The post-transcriptional regulation of DASPO expression has been investigated less and little is known about the encoding transcript stability and protein synthesis levels. Intriguingly, a preliminary in silico analysis of the 3′-UTR sequence of both *DDO* and *DAO* genes very recently disclosed the occurrence of several potential binding sites for microRNAs that are expressed in the CNS and that are known to bind sequences conserved in mammals (Errico et al., 2020). However, only few of the predicted binding sites were concomitantly present in human and rodents, weakening their potential as gene expression regulatory mechanisms. Moreover, the lack of post-translational modification studies on hDASPO represents a current research gap.

## Single Nucleotide Polymorphisms and Missense Variants

In their study, the work of Usiello and his group proposed that two intronic SNPs within the human *DDO* gene, namely, rs2057149 (A/G) and rs3757351 (C/T), have a functional role in modulating hDASPO levels in the prefrontal cortex of healthy individuals (Errico et al., 2014). The two SNPs are associated with *DDO* mRNA expression: the A and C alleles predict reduced transcription compared to the G and T alleles, respectively. Moreover, based on brain imaging analysis, the authors proposed that this genetically mediated decrease in hDASPO expression, mapped on the prefrontal phenotype, suggests that neuronal plasticity increased and neural networks were activated during working memory processing. Accordingly, the C allele of rs3757351 is also associated with a greater prefrontal gray matter volume and higher activity during this task than is the T allele (Errico et al., 2014). Additionally, the human gene database GeneCards (https://www.genecards.org/) reports three SNPs in the *DDO* gene coding sequence that present missense substitutions with no clinical significance: rs17621, rs17622, and rs17623 coding for the His230Tyr, Gln189Glu, and Leu255Arg substitutions, respectively (numbering refers to the canonical protein isoform, hDASPO_341). Interestingly, Gln189 and Leu255 are highly conserved in mammalian DASPOs but not in those of rodents, while His230 is a poorly conserved residue. These residues are located on the protein surface and the indicated substitutions probably only poorly affect the enzyme properties.

Conversely, the missense variant VAR_036244 (Phe136Leu in hDASPO_341) is reported in GeneCards as a somatic mutation in a breast cancer sample. The substituted residue is part of the *β*-strand 131–141 and is strictly conserved in all mammalian DASPOs. Finally, the catalogue of somatic mutations in cancer (COSMIC, https://cancer.sanger.ac.uk/cosmic) reports another missense variant as being associated with esophageal cancer, i.e., the Gly337Arg substitution in the hDASPO_369 protein isoform (corresponding to Gly309 in hDASPO_341 isoform). This Gly residue belongs to a short amino acidic sequence (HHYGHGSGG) strictly conserved in all mammalian DASPOs (and hDASPO protein isoforms). Notably, both Phe136 and Gly309 are close to the active site (Figure 5A): in particular, the Gly309Arg substitution might deeply impact enzyme function. The COSMIC database reveals several hDAAO somatic variants identified in tumor tissues: the actual effects of the reported amino acidic substitution have not been investigated yet.

A query using the ClinVar tool at the NCBI site (www.ncbi.nlm.nih.gov/clinvar/) indicates that there are no clear relationships among missense mutations in *DDO* gene and human phenotypes associated with other human pathologies. All the additional SNPs currently present at the UCSC Genome Browser site (https://genome.ucsc.edu/) introduce sequence variations at noncoding regions of the *DDO* gene: they represent 3′-UTR, intron, and 5′-UTR variants whose effect is unknown.

## D-aspartate Oxidase vs. D-Amino Acid Oxidase

From a biochemical point of view, hDASPO and hDAAO significantly differ in several aspects, suggesting that, during evolution, two different ways emerged to modulate D-Asp and D-Ser levels in the brain.

hDASPO and hDAAO show a different pH profile while activity and stability dependence on temperature are similar (Katane et al., 2015a; Murtas et al., 2017). In solution, hDASPO is monomeric while hDAAO is a homodimer. A dimeric quaternary arrangement (similar to the one observed in hDAAO) is apparent only when hDASPO is packed into the crystal asymmetric unit: under these conditions, the stabilization of the oligomeric form is due to the presence of a phosphate and a Tris ion that mediate the monomer-monomer interactions at the dimer interface located in the apical region of the SBD. The space occupied by the phosphate and the Tris ions at the interface between monomers in hDASPO is filled by the side chains of two residues (Phe133 and Lys211) directly involved in protein-protein contacts in hDAAO (Molla et al., 2020). The different oligomeric state between hDASPO and hDAAO in solution is due to differences at the dimerization interface: Asp73, His80, Phe90, Arg120, Met124, Phe133, and Lys211 residues in hDAAO are replaced by Asn73, Asn80, His90, Ala122, Lys126, Ala135, and Glu212 in hDASPO. This results in an alteration of the overall hydrophobicity and polarity of this region. Notably, the different oligomerization state could affect subcellular trafficking and the modulation by interacting proteins (Kawazoe et al., 2006; Molla et al., 2006; Molla et al., 2020).

According to the manifold physiological roles, hDAAO shows a wide substrate acceptance: the best substrates are hydrophobic and bulky d-amino acids, but it is also active on small, uncharged ones. Conversely, hDASPO is highly specific for acidic substrates. At both active sites, the *α*-carboxylate of the substrate interacts with a Tyr and an Arg residue (Tyr223 and Arg278 in hDASPO; Tyr228 and Arg283 in hDAAO) while the side chain is accommodated in a pocket with a different chemical environment (Kawazoe et al., 2006; Molla et al., 2020). In hDASPO, this active-site pocket is positively charged and constituted by loop 217–221 and His54, Arg216, and Arg237; the same pocket in hDAAO is made up of bulky and hydrophobic residues (Leu51, Gln53, Leu215, and Ile230) (Kawazoe et al., 2006). Moreover, the conformational change of loop 216–228 and, in particular of Tyr224, allows hDAAO to bind larger substrates (Kawazoe et al., 2006; Molla et al., 2006). On the other hand, this loop is six residues shorter (loop 217–221) in hDASPO and only partially shapes the active site entrance (Figure 5). Indeed, two additional loops in this region (namely, 54–63 and 101–107) show a significant conformational difference in comparison with hDAAO. These differences contribute to increasing the hDASPO turnover number compared to hDAAO (Molla et al., 2020). Actually, both flavoenzymes follow a ternary complex mechanism but with a different rate-limiting step in catalysis: it is the product release in hDAAO (<1 s^−1^, since the conformational change of the active site lid is relatively slow) (Molla et al., 2006) and the step of reoxidation of the reduced flavin in hDASPO, see above (Molla et al., 2020). In line with this evidence, hDASPO shows on D-Asp a 10-fold higher specific activity than hDAAO on D-Ser (Molla et al., 2006; Molla et al., 2020). K_m_ and K_d_ values for the best substrates are in the millimolar range for both flavoenzymes, suggesting that under physiological conditions the enzymatic activity is strongly affected by the substrate concentrations (Molla et al., 2006; Molla et al., 2020). The variations in the active site architecture are also responsible for the lower affinity observed for hDASPO with CBIO and DPPD in comparison to hDAAO (Table 3): a partial steric hindrance from Ser308 and Ile52 should hamper the positioning of the rigid aromatic ring of the inhibitor parallel to the isoalloxazine ring of FAD (Molla et al., 2020).

No substantial differences in the mode of interaction of the cofactor with the protein moiety are apparent between the two human flavoproteins (Molla et al., 2006; Molla et al., 2020). The sole significant difference is the replacement of Trp185 in hDAAO by Gly186 in hDASPO in the region of the protein close to the FAD ribose moiety. Thus, the different affinity for the cofactor between the two flavoenzymes is probably due to different dynamics during the flavin binding and release processes. hDASPO does not seem to be modulated *in vivo* by ligand and flavin binding: its interaction with FAD is very strong (K_d_ in the nanomolar range vs. a figure of 8.0 *μ*M for hDAAO) (Molla et al., 2020). Owing to the weak interaction with FAD, hDAAO is present in solution as an equilibrium of holo- and apoprotein forms at the concentration of the cofactor in brain (2–5 *μ*M) (Caldinelli et al., 2010; Murtas et al., 2017), as also reported for mDASPO (Puggioni et al., 2020), while hDASPO is fully present as active holoenzyme (Molla et al., 2020).

## Conclusion and Future Perspectives

The d-enantiomer of aspartate presents many of the signatures of a classical neurotransmitter and in recent years has attracted attention for its involvement in main physiological processes. Here, recent investigations of the structure-function relationships in hDASPO are shedding light on its properties and highlighting the differences with the homologous flavoenzyme hDAAO, too. Notwithstanding a high degree of sequence and structure conservation, evolution diverged to produce amino acid oxidases that control the brain concentration of the neuromodulators D-Asp and D-Ser differently. While D-Ser levels must be maintained in a physiological range to avoid NMDA receptor hypofunction (thus, the weak activity of hDAAO is only sufficient to avoid an accumulation of D-Ser), the D-Asp levels seem to be tightly controlled by hDASPO, as observed during neurodevelopment. Such a high enzymatic efficiency might be responsible for negative outcomes since the dysfunctional D-Asp metabolism occurring during neurodevelopment may affect early critical processes related to NMDA receptors (Errico et al., 2018). For example, disrupted D-Asp metabolism (increased levels in the prefrontal cortex, hippocampus, and serum) was recently reported in BTBR mice, an animal model of idiopathic autism spectrum disorder (Nuzzo et al., 2020). A dysfunction in NMDA receptor-mediated neurotransmission due to decreased D-Asp levels in the nervous system is thought to occur during the onset of various mental disorders, including schizophrenia (Nuzzo et al., 2017; Errico et al., 2018). In this regard, treatment aimed at increasing D-Asp levels (and thus at activating NMDA receptor function) represents a novel and useful therapy. Such a relevant goal can be reached by acting on hDASPO: the inhibition of the enzyme activity would prevent D-Asp degradation. Known hDASPO inhibitors possess a low *in vitro* potency and thus may represent lead compounds for the development of new drugs based on a rational, structure-guided design, taking into account the differences in the active site geometry with hDAAO. Such molecules can be also useful to treat infertility since D-Asp is thought to be involved in the quality control of germ cells and to stimulate myelin repair in the disability associated with multiple sclerosis.

A main controversy in the field is related to the biosynthetic D-Asp pathway in human brain: the presence of a synthetic enzyme is strongly considered (both an enzyme acting similarly to serine racemase for D-Ser or using a completely different mechanism) but its identity is still elusive, which will attract the attention of researchers in the near future. Indeed, forthcoming studies also need to focus on the modulation of hDASPO function by post-translational modifications, protein interaction, and cell targeting under both physiological and pathological conditions.
